# Acoustic Emission in Bone Biomechanics: A Comprehensive Review of Mechanical Properties and Predictive Damage Modeling

**DOI:** 10.3390/s25030598

**Published:** 2025-01-21

**Authors:** Silvia García-Vilana, David Sánchez-Molina, Hamed Abdi

**Affiliations:** 1 Escola Politècnica d’Enginyeria de Vilanova i la Geltrú (EPSEVG-GiES), Universitat Politècnica de Catalunya, Víctor Balaguer, 1, 08800 Vilanova i la Geltrú, Spain; silvia.garcia.vilana@upc.edu; 2 Escola d’Enginyeria de Barcelona Est (EEBE), Universitat Politècnica de Catalunya, Av. Eduard Maristany, 16, 08019 Barcelona, Spain; 3 Sina Trauma and Surgery Research Center, Tehran University of Medical Sciences, Tehran 11365-3876, Iran; h-abdi@farabi.tums.ac.ir

**Keywords:** acoustic emission, bone mechanics, non-destructive evaluation, fracture prediction, micro-damage modeling

## Abstract

This review investigates the application of acoustic emission (AE) techniques in analyzing the mechanical properties and damage prediction of human bone. AE is a non-invasive and non-destructive evaluation method that captures the elastic waves released during microstructural deformations under stress, providing critical insights into bone behavior and failure mechanisms. By analyzing 57 studies, this review synthesizes findings on AE signal characteristics, experimental configurations, and their correlations with bone’s mechanical parameters such as yield strength, elastic modulus, and micro-damage evolution. This article highlights AE’s potential in early damage detection, differentiation of failure modes, and predictive modeling using stochastic and percolation theories. These models facilitate the prediction of fracture risk and mechanical failure without inducing irreversible damage. This review addresses the strengths and limitations of AE techniques and outlines future directions in biomechanical research.

## 1. Introduction

Non-invasive techniques can provide information on crack initiation that histology cannot measure [1,2]. More specifically, acoustic emission (AE) is a non-destructive evaluation method that operates without introducing external energy into the material being studied [3]. More recently, the quantitative analysis of AE signals by means of principal component analysis has made it possible to associate specific types of micro-failures inside brittle materials; thus, matrix cracking, fiber-matrix debonding, delamination, and fiber breakage can be distinguished by accurate analysis of AE signals [4].

AE is based on the principle that, when internal stress acts on a material, its micro-structure deforms, storing the applied mechanical work as elastic potential energy. Due to the inherent heterogeneity of bone’s micro-structure, stress concentrations develop at specific microscopic sites. When these stress concentrations exceed a critical threshold, they cause localized micro-damage within the micro-structure [5]. Micro-cracking reduces internal stress in the affected areas, releasing some of the stored elastic potential energy as elastic waves [6,7,8,9]. These elastic waves manifest as spontaneous ultrasonic signals originating from the stress concentration sites. By triangulating these signals using multiple AE sensors, one can estimate the location and severity of the micro-defects based on the total recorded energy [3,10].

Each detected AE signal has specific characteristics that provide information about the type of event that produced it. On one hand, the energy of the AE signal is the area under the squared waveform curve, and the amplitude represents the strength of the signal. The *rise time* is the time elapsed from the start of the event, above the threshold (noise limit), to the signal peak. The *rise time over amplitude* is the ratio of *rise time* to amplitude, and the average frequency refers to the number of times the signal exceeds the threshold relative to its duration [11]. These signals are found within frequencies ranging from 150 to 300 kHz, above the audible sound range [9].

Multiple techniques have been utilized in the literature to evaluate the condition of bone, such as mechanical impedance, natural frequency, wave propagation, ultrasonic measurements, and impact response techniques. Among these, AE has also been used, proving to be a valuable tool due to the similarity between bone and composite materials [12]. It is worth noting that the hierarchical structure of bone is fundamental to its mechanical functionality and the characteristics of AE. It has been highlighted how bone’s complex architecture, spanning nano- to macroscales, underpins its exceptional mechanical performance, including high strength and fracture point [13]. Building on this, Reznikov et al. (2014) used advanced imaging to detail the three-dimensional organization of collagen fibers, mineral crystals, and lamellar units, illustrating the multiscale interplay between structure and function [14]. Together, these studies emphasize bone’s optimized design as a natural material that balances strength, flexibility, and biological adaptability.

The goal of this review article is to summarize the use of AE in human bone as reported in the literature. It explains the type of information that can be extracted using AE, compares the results of different experimental configurations, and outlines the advantages and limitations of each approach employed. This article concludes with an in-depth examination of quantitative models that enable predictions near the point of fracture.

## 2. Selection Criteria for Reviewed Articles

One of the aims of this review is to describe the use of AE as a technique for predicting the mechanical behavior of bone. We included studies that examine AE applications in bone related to mechanical parameters such as maximum force and elongation, mechanical properties like ultimate strength, deformation, yield strength, and elastic modulus, as well as bone micro-cracking or damage. Additionally, we considered articles that develop predictive models or equations of mechanical behavior based on AE.

**Figure 1:**
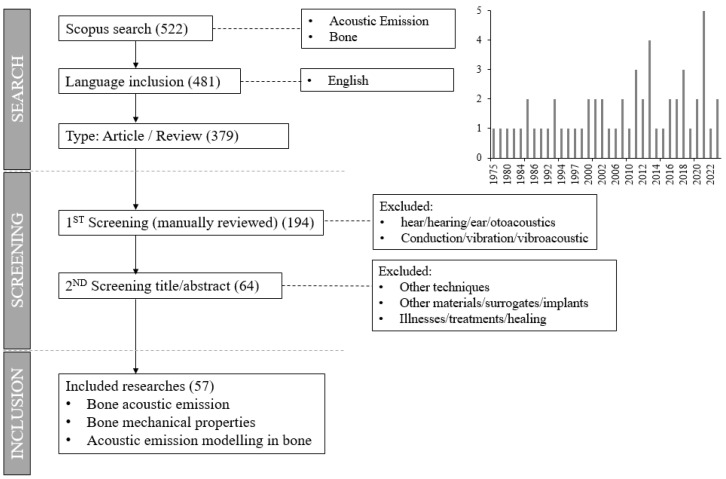
Scheme of selection process with refinement of criteria for inclusion and exclusion.

The article search was divided in three stages. The search stage was performed in the Scopus database, encompassing all articles published up to 24 May 2024 which included the terms “acoustic”, “emission”, and “bone”, identifying 522 documents. From those, 143 documents were automatically excluded due to language restrictions (only English was considered) or because they were documents different than articles or reviews (conference papers, book chapters, or others). In the second stage, an automatic screening was performed based on the terms “ear”, “hearing”, or “hear”, identifying 185 articles related to otoacoustics or other applications falling outside the scope (the screening was manually reviewed reading all abstracts). The abstracts of the remaining articles were read to exclude those of other topics such as bone machining, illnesses, other materials or techniques, or implants and surrogates, identifying 130 articles to exclude. In the last stage, the remaining 64 articles were read, and from those, 7 were excluded for being outside the scope. Thus, 57 articles and reviews were considered, which use AE to evaluate bone performance and mechanical parameters or to develop models. The selection process, including the refinement of criteria and the temporal distribution of the analyzed studies, is detailed in Figure 1 and explained in more detail in Appendix A.

While this review focuses on studies that have used AE to analyze mechanical properties, it is important to acknowledge the existence of prior reviews that have also examined aspects related to this issue [15,16,17,18].

## 3. Use of AE in Human Bone Research

AE techniques have been widely employed in bone studies, in areas ranging from the detection of injuries and pathologies such as osteoporosis or osteopenia, to identifying the optimal timing for fixation removal [19], as well as for implant insertion or assessing the effects of gamma radiation [20]. Table 1 provides a general panorama for the use of AE to investigate human bone.

**Figure 2:**
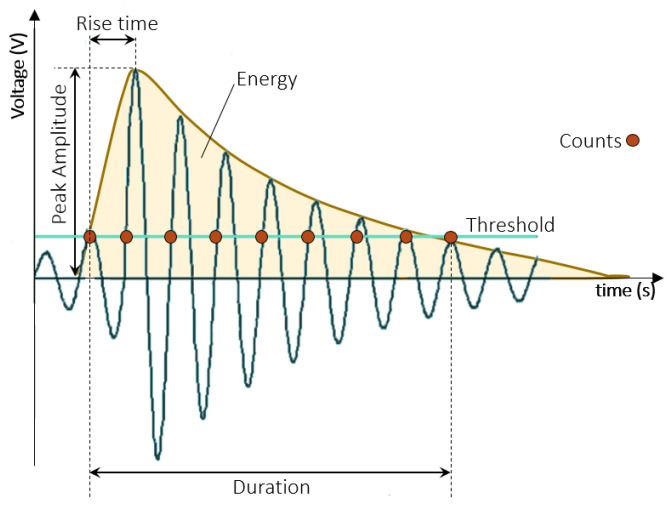
Key concepts in AE signals: *Peak amplitude* refers to the maximum amplitude of the wave. *Rise time* is the interval between the moment the amplitude exceeds the threshold and when it reaches its peak amplitude (waves with amplitudes below the *threshold* are ignored). The *count* magnitude indicates the number of times the wave surpasses the threshold. *Duration* is the time interval between the first hit and the last hit. Finally, energy is approximately calculated from the wave’s envelope.

The quantity and qualitative characteristics of AE signals are correlated with the mechanical properties of bone, as well as with its structural behavior during the damage process induced by mechanical loading [21]. The use of AE allows for the differentiation between mechanical failures of various biological tissues. For instance, the study by VanToen et al. (2012) subjected a set of human vertebrae along with their ligaments to testing, observing higher-amplitude peaks in vertebrae than in ligaments, as well as AE signal avalanches occurring near the failure point; the differences were attributed to the distinct failure mechanisms in these components [22]. In addition, the specific type of micro-failure can be associated with many specific signals [4]. The key concepts of AE measurement are detailed in Figure 2.

Furthermore, detecting and localizing the origin of AE signals allows for the anticipation of the region and stress level at which a fracture might occur or the area experiencing deformations [23]. For this reason, several studies have focused on proposing models or methodologies for localizing fractures detected via AE, such as through multiple regression analysis [23] or hyperbolic localization [24]. Both methods demonstrate the possibility of detecting and localizing AE signals before a fracture occurs [25]. The propagation of AE signals has also been shown to depend on the level of dehydration in tests involving wet and dry bovine bone [26]. In addition, some studies have conducted comparisons of fracture detection in medical images obtained by computerized tomography (CT) and AE, while microCT has been used to visualize bone fracture using incremental quasi-static loading, as three-dimensional imaging sequences are not yet capable of acquiring the temporal characteristics of a bone fracture [27,28].

AE has also been implemented to characterize the mechanical behavior of cortical bone, mainly with the goal of correlating AE signals with the failure mechanisms [18]. The first investigation using AE to study the mechanical properties of bone was conducted by Hanagud et al. (1974) [29]. This study analyzed differences in AE signals between intact bone specimens and those with pre-existing cracks, both subjected to bending, detecting AE signals earlier and with greater intensity in cracked bones. These findings already indicated differences in damage propagation between intact and pre-cracked samples. Subsequent studies have demonstrated how AE use in bone allows for differentiation between stages of bone behavior. The study by Knet et al. (1975) conducted tensile tests on cortical femur bone and clearly identified three stages based on AE signals: (1) an initial stage up to 0.21% strain relative to failure, where only 2% of AEs were detected with a non-linear increase, (2) a second stage from 0.21% to 0.87% strain with a linear increase between *N* and ε, an event rate of 20 hits/s, and 46% of cumulative hits, and (3) a final stage until failure, characterized by a large avalanche of hits and a pronounced increase in micro-cracks, with a rate of 210 pulses/s [30]. The similarity between the stress–event and stress–elastic energy curves, along with the high correlation coefficient between events and energy ($r^{2}=0.93$), clearly demonstrated the energetic nature of AE. Additionally, the near absence of events in the first stage was attributed to the Kaiser effect (the inability to detect events for repeated loads that do not exceed the previously applied maximum load). This effect was corroborated in a later study by Krauya et al. (1978) in fatigue tests with progressive load increments on human tibiae. The authors observed that more emissions were detected during the first load of each cycle than in subsequent loads at the same force level. They also detected a negative correlation between strain and AE signals. This demonstrates the irreversibility of processes in bone beyond a certain deformation level, which is detected by AE, where relaxation and reloading at the same force level practically do not increase the number of hits [31].

**Table 1:** Relations of AE, load, and other variables found in the literature. (AE = number of acoustic emission events, RT = rise time, RA = rise time over amplitude, NS = non significant).

Study	Results
AE detection-strain [30]	*Tensile tests*. Three AE stages: - $\varepsilon /\varepsilon_{\max}<0.21$ (less AE) - $0.21<\varepsilon /\varepsilon_{\max}<0.87$ (20 pulses/s) - $0.87<\varepsilon /\varepsilon_{\max}<1$ (210 pulses/s)
AE detection-load [9,32,33,34,35]	*Tensile:* - Linear relation between load AE onset and AE events *Torsion:* - Fracture 30–50% total twist (tibia/femora dog) *Bending-torsion:* - AE onset 6.7–38.3% load (human femoral head) - 89% AE events belong to torsion - Shear stress: longer RT and RA than normal stress - AE activity at 1/7 maximum load *Fatigue:* - AE onset at 95% fatigue life *Compression* (trabecular): - AE onset 30–40% maximum load - AE in linear behavior (different than compact bone)
Bone pathology [22,36,37,38]	*Osteoporotic/Osteoarthritic/Osteopenic:* - Events under yielding/higher amplitude - Higher cumulative AE and at lower strain/load - More microdamage at early stages
AE-damage [9,20,39]	Specimens with *notch:* - Higher energy in transverse - Early AE activity stage Higher cumulative AE Correlation AE–damaged area Higher amplitudes in damaged specimens
AE-treatment [19,20,40]	- *Gamma radiation:* AE decrease (12–46%, less damage) - *Fixations:* higher AE onset load with healing time - *Immobilization:* higher AE (lower mechanical properties, NS)

However, when analyzing the literature on mechanical characterization, it becomes evident that AE usage has been mainly limited to detecting the onset of bone failure or examining how the number of emissions and their initiation vary across different types of tests. While these studies have shown AE’s great potential for failure detection, this approach, while useful for studying differences between materials or testing conditions, does not allow for the prediction of a material’s fracture stress. This limitation arises from the high variability in the maximum force or displacement values exhibited by biological materials like bone, due to factors such as geometry, anthropometric variables like age or sex, the subject’s physical condition, and the random presence of micro-defects that act as stress concentrators in the material. The need for genuinely predictive models capable of anticipating fracture stress requires establishing a quantitative relationship between the parameters of the random distribution of AE signals and the intrinsic mechanical properties of bone, such as maximum strength, maximum strain, or yield limit. However, only a small fraction of studies have addressed the challenge of providing predictive models that link AE with structural failure in bone. These models are of particular interest in non-destructive techniques, which aim to predict material failure without causing severely irreversible damage.

### 3.1. Damage Detection and Failure Mode Characterization

Damage in bone or micro-defects that lead to friction or elastic energy dissipation generate detectable signals via AE. Analyzing these signals enables the identification of the specific types of micro-failures they correspond to [4]. The potential to understand bone failure mechanisms through AE was demonstrated decades ago in a pioneering study by Carter and Hayes (1977), which showed a clear association between non-linearity (or the onset of the non-elastic region) and the level of micro-cracking [41,42]. This work already indicated the possibility, later confirmed, that AE could be a highly valuable technique for detecting bone damage. Indeed, AE has proven to be an extremely useful tool in analyzing the different failure modes occurring in bone. In particular, AE can be employed for the early detection of micro-structural failures, even before micro-cracks can be observed through micro-radiography [37], making AE a highly capable technique. For example, in studies such as that of Aggelis et al. (2015), where flexural–torsional tests were performed on the human femoral head, AE signals revealed that micro-cracking begins at a force as low as one-seventh of the maximum [32]. Furthermore, as a crack propagates, AE signals are detected in bursts distributed at irregular (random) intervals, which are attributed to the significant amount of energy involved in the cracking process [36]. It is also well-known that AE signals become detectable when the applied force reaches the bone’s non-linear behavior region [37], occurring before the maximum force, at which cracks begin to initiate [39]. Several studies have focused on locating damage using signals collected from multiple sensors [43].

In addition to early damage detection, AE has also proven useful for identifying the mode by which cracks propagate through the bone structure. The study by Akkus et al. (2000) analyzed emissions associated with cracks propagating longitudinally and transversely to the osteon orientation during tensile tests on human femoral bone. The more abrupt force drop observed in transverse specimens was found to correlate with the sharp increase in AE signals, while in longitudinal specimens, the number of signals grew more gradually. Additionally, damage modes were differentiated based on energy and signal count: (1) the propagation of the main crack, characterized by a few high-energy signals; and (2) diffuse damage and secondary micro-cracking, characterized by a large number of low-energy signals. The highest-energy events were observed in transverse cracking, whereas less energy was required for longitudinal crack propagation [39]. The relationship between damage and the number of AE signals was further corroborated in a later study by Akkus and Rimnac (2001), where the area ratio of damaged regions (%AR) in human femoral cortical bone was calculated by dividing the section into a grid and counting the damaged squares. This %AR ratio was found to be highly correlated with the total number of emissions detected [20].

### 3.2. Amplitude and Number of AE Signals

An important characteristic of AE signals is their amplitude, which is correlated with the damage of a micro-fracture event or, equivalently, the energy released, making it a generally important parameter [32]. There is a direct correlation between signal amplitude, energy release, and the resulting damage magnitude. Subsequent studies, such as that by Fischer et al. (1986), have confirmed the dependence of AE on strain rate. In their research involving tensile tests on bovine cortical bone, signals were initiated near the point of failure, and a greater number of lower-amplitude signals were observed at slower strain rates. The amplitude of these events was characterized using the peak amplitude *V* and a threshold $V_{0}$ with the relation $F\left(V\right)=F\left(V_{0}\right){\left(V/V_{0}\right)}^{-b}$. Here, the parameter *b* indicates the distribution of event amplitudes—higher *b* values signify a larger proportion of low-amplitude events, with observed values ranging from 0.67 to 1.01. Specimens tested at lower speeds exhibited higher *b* values, and events initiated near the fracture point. This suggests that bone behavior under dynamic loading resembles that of brittle materials, which display high-amplitude events. It also implies that the AE mechanisms in bone under tension align with seismological models like the Gutenberg–Richter law [11]. Similarly, VanToen et al. (2012) observed higher amplitude peaks under dynamic loading of vertebral bodies compared to quasi-static conditions [22].

AE has been effectively used to study bone fractures in various contexts. In impact studies on nasal bone and frontal lobe, AE was utilized to predict the force initiating fractures, allowing for the development of risk curves for these bones [44,45]. Additionally, tests on femurs from elderly subjects involved applying flexo-torsion loads using a piston on the femoral head. Emissions were primarily detected between 10 and 15% of the maximum force, with a higher ratio of high-energy emissions in specimens with thicker cortical layers. Notably, 89% of the high RA signals corresponded to torsion fractures, indicating that shear stresses exhibit higher RT and RA compared to bending stresses (tensile stresses above and compressive stresses below the neck) [32].

In compression tests on bovine trabecular bone, AE signals were detected on average at 36.6% of the maximum force, mostly between 30 and 40% [33]. The study did not find clear correlations between material parameters and AE parameters, possibly because some specimens failed destructively while others did not. Unlike cortical bone, trabecular bone exhibits purely linear elastic behavior according to this research [33,46]. As cited by Agcaoglu and Akkus, trabecular bone shows wider ranges of AE due to different failure patterns, with emissions primarily occurring in the non-linear range and increasing asymptotically near fracture [34,47].

Karaduman et al. (2018) conducted tensile tests on bovine tibia metaphysis samples, some with pre-existing cracks of 6 and 12 mm and others intact [9]. They identified three AE regions: initiation, low emissions, and very high emissions during catastrophic failure, observing a linear relationship between the force at initiation and signal generation. Crack initiation occurred between 75.8 and 94.4% of the maximum force, suggesting that emissions below this range are not significant for remodeling.

### 3.3. Mineralization, Anisotropy, and Porosity

Mineralization, assessed through bone mineral density or bone mineral fraction, is a key determinant of bone stiffness and strength, which influences the characteristics of the detected AE signals. Understanding the exact relationship between mineralization and AE signal quality requires detailed signal classification based on specific features—a method not commonly applied in most studies. While anisotropy is also expected to play a role, the energy of AE signals does not appear to be affected. Nonetheless, other signal characteristics, which have not been explored in the current literature, might show a detectable influence. Table 2 summarizes studies investigating these factors in relation to AE. Trȩbacz et al. (2011) analyzed differences in AE between fresh and demineralized bovine cortical bone subjected to compression with up to 3 mm of deformation. They observed that demineralized bone exhibited AE signals throughout the entire deformation range with much lower energy. In contrast, fresh bone showed AE signals only at the onset of the non-linear deformation range and at the end of the test. Additionally, samples deformed longitudinally displayed lower accumulated energy and less energetic signals at higher deformations. These directional differences are attributed to variations in the alignment of collagen lamellae and fibers [7].

Further studies suggest a relationship between porosity and AE signals. Anteromedial and posterolateral samples from the diaphysis of bovine femoral bone were collected; half were subjected to 300 cycles of three-point bending. All samples were demineralized and cut into cubes for compression testing. The anteromedial samples exhibited a lower fraction of large pores and correspondingly lower emission levels. Conversely, the posterolateral samples had higher AE activity and a greater percentage of large pores [48].

Loading rate has also been shown to influence detected AE events. Fischer et al. (1986) found more AE events with lower amplitude with higher strain rate in fresh bovine cortical bone under tensile tests [11]. Moreover, Moiseev (2016) performed compression tests on vertebral segments—including vertebrae, discs, and ligament—under quasi-static and dynamic loading conditions. The results indicated that dynamic loading produced a greater cumulative number of AE signals and that damage appeared at lower load and deformation values compared to the static case (though the latter was not statistically significant) [49]. Thus, for a given axial load, trauma is greater when the load is dynamic.

Specimens with higher levels of mineralization produced fewer AE events, suggesting that bone specimens with greater mineral content exhibit less acoustic activity but enhance fatigue resistance by increasing the energy required for cracks to propagate; hence, more energy per emission is observed. However, fatigue life did not correlate with mineral content. Mineralization is inversely related to the time to failure after the first emission—meaning that specimens with more mineral content took longer to exhibit the first emission and thus fail in a brittle manner [34].

### 3.4. Correlation with Mechanical Properties

The most commonly measured mechanical properties include the elastic modulus, which reflects stiffness; the yield strength, which describes post-elastic behavior; and the ultimate stress, at which the highest concentration of AE signals is typically observed. These properties have been systematically analyzed alongside the distribution of AE signals relative to stress or strain to uncover meaningful correlations. But determining the inherent mechanical properties of human cortical bone, especially when incorporating AE techniques during mechanical testing, presents specific challenges. Firstly, limitations exist in performing mechanical tests on bone obtained from deceased individuals. Secondly, the bone’s geometry—with variations in thickness, cross-sectional area, and occasional curvatures—complicates its characterization and affects sensor stability [32]. Due to these complexities, approximations are frequently employed in calculating bone mechanical properties. For instance, when calculating stresses, specimens are often assumed to have solid and homogeneous areas, neglecting porosity or density variations. The geometry may be approximated to a simple, known shape, or in bending tests, beam theory is used with a focus on the central section, which clearly influences the second moment of area of the cross-section. Similarly, strain is sometimes determined from the displacement of the fixtures or by using bending standards that rely on common non-biological linear approximations, which are inadequate for large strain situations. One of the most accurate techniques for determining strain is digital image correlation (DIC). A study from the past decade showed that, in bovine trabecular bone, using crosshead displacement to calculate the elastic modulus from the relative displacement of plates underestimates this parameter by up to 23% compared to its determination in a small central area using DIC [33].

Previous studies, such as those by Leichter et al. (1990), have found a relationship between mechanical properties and the characteristics of AE signals in mechanical tests on trabecular bone from the femoral heads of healthy subjects, as well as those with osteoporosis and osteoarthritis. It is known that bones affected by these pathologies exhibit degraded mechanical properties, such as reduced elastic modulus, yield strength, and ultimate strength. In their study, a higher rate of AE signal production and greater signal amplitudes were observed in bones from subjects with pathologies, attributed to the fragility and thinness of the trabeculae. In all cases, the rate of detected events was notably higher above the yield strength [36]. However, despite the relationship between signal amplitude and the energy released during fracture—which is somewhat contradictory in the findings of this study—the authors attribute this to external factors influencing amplitude, such as attenuation due to increased porosity.

Similarly, subsequent research by Hasegawa et al. (1993) observed more emissions at low forces in vertebrae from women with osteopenia compared to those from healthy subjects (all specimens were embalmed), and fewer emissions in the non-linear region. They deduced a higher rate of accumulated micro-damage in the early stages of deformation in bones with pathologies. This indicates that stress distribution is more uniform in healthy bones, which reach failure immediately with a massive amount of micro-damage once the maximum force is attained [38].

It is noteworthy that various types of mechanical tests have been conducted in studies, including tensile tests [12,30,31,50], simple compression tests [33,36,49,51,52,53], three- and four-point bending tests [9,29,34,40,54], and torsion tests [35,55]. However, none of the studies cited focused on analyzing the relation of commonly used intrinsic mechanical properties (i.e., maximum stress or strength, maximum strain, Young’s Modulus) with the AE characteristics, its cumulative number, or similar features, which impedes the understanding of the influence of bone tissue mechanical behavior (without the influence of the bone geometry used) on AE response variables.

## 4. Predictive Models Based on AE

The anticipation of mechanical failures in bone tissue under applied stress represents a topic of significant practical relevance [56,57,58]. Unfortunately, many studies on AE focus solely on the applied forces and observed displacements in specimens. However, because these quantities are not intrinsic variables—since factors such as specimen length and cross-sectional area are not accounted for—the predictive power of such studies is inherently limited. Research has consistently shown that micro-defects and the stochastic characteristics of bone micro-structure play a critical role in the initiation of micro-cracks and the subsequent development of macroscopic fractures [59,60,61,62].

Although there is a statistical correlation between stress and strain within the bone and the occurrence of micro-failures and the generation of AE signals, the process of micro-damage production depends on random details of the micro-structure. Consequently, it is generally not possible to formulate a completely deterministic model of the appearance of AE signals. For this reason, several authors have resorted to stochastic methods in reliability theory to model the progression of damage [63]. Such methods can be used to study cumulative damage processes based on the Poisson shock model [64] and Markov chains [65,66]. Table 3 compares the models used for predicting bone fractures.

### 4.1. Failure Models and Reliability Theory

A way to use AE data is the development of risk curves for bone fracture [44,45,52]. However, these models rely solely on a posteriori analyses and generally do not offer precise predictions regarding the likelihood of new micro-failures. Instead, they provide a global estimation of failure probability, typically expressed in terms of a risk index that is often related to an applied force. An alternative approach involves the use of probabilistic models that take into account the accumulation of previous micro-damage, since successive AE signals do not occur independently. In fact, the presence of micro-damage in a given region increases the likelihood of additional AE signals appearing due to localized bone weakening. Thus, we consider a *stochastic process* formed by a collection of random variables ${N_{\varepsilon }}_{\varepsilon \geq 0}$, in which the number of detected signals is a random variable dependent on the longitudinal strain ε. The number $N_{\varepsilon }$ would be correlated with the number of AE events experimentally detected [62,65]. In this type of models, AE signals occur at random over the loading history in accordance with a non-homogeneous renewal Markov stochastic process. Let $\varepsilon_{k}$ denote the strain when the *k*-th micro-failure occurs, and let $\Delta \varepsilon_{k+1}$ be the strain increment between the *k*-th and (k+1)-th micro-failures. Due to the material degradation from each micro-failure, the expected strain intervals decrease:

(1) $$E\left(\Delta \varepsilon_{k}\right)\geq E\left(\Delta \varepsilon_{k+1}\right).$$

As the material approaches failure, micro-failures accumulate rapidly. By making different hypotheses about how the occurrence of one micro-failure affects the next, various models can be developed. For example, several models used in biological tissues assume that the increment in strain or stress between micro-failures follows an exponential distribution $\Delta \varepsilon_{k}∼\mathrm{Exp}\left(\lambda_{k}\right)$ (as in continuous-time Markov chains). In this case, the expected value between micro-failures depends on the number *k* of previous micro-failures, that is, $E\left(\Delta \varepsilon_{k}\right)=1/\lambda_{k}$ [62,65]. The exponential distribution is chosen for its memoryless property. In this family of models, $\lambda_{k}$ is a decreasing function of *k* due to material embrittlement. A particularly simple choice is the following:

(2) $$\lambda_{k}=\lambda \alpha^{-k+1},\;\alpha <1,$$

ensuring the process leads to failure at a finite strain, consistent with experimental observations. The deterioration rate in this case is determined by the reduction in the observed intervals between AE signals:

(3) $$\beta_{k}=-\ln\frac{\lambda_{k+1}}{\lambda_{k}}>0,$$

**Figure 3:**
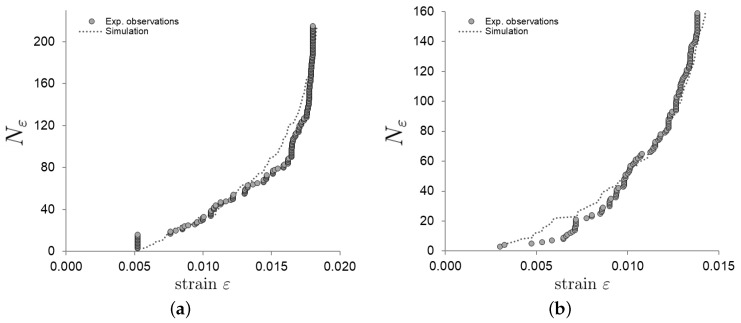
Comparison of the AE events observed and simulated as a function of strain ε, for two specimens (**a**,**b**) (adapted from [62]). In each of the two graphs, the experimental data are represented by gray circles. These observations are used to estimate the model’s parameters, which are then applied to simulate corresponding curves depicted by dashed lines. As can be seen, AE signals (hits) near the maximum stress form a vertical asymptote, because the deterioration parameter leads to an accelerated damage accumulation.

It can be observed that each instance of the aforementioned stochastic process generates a possible sequence of AE signal occurrences. By comparing the observed AE signal sequence as a function of the measured strain, one can then fit the values of the degradation factor (α) and the signal interval for intact bone ($\lambda_{0}$) (see Figure 3).

### 4.2. Failure Models and Percolation Theory

Another possibility in predictive models, based on probabilistic ideas, is the use of percolation theory models. In general terms, percolation theory examines how connectivity networks emerge in random systems as the probability of element occupation increases. In the context of mineralized collagen fibrils, this theory is used to understand how mineral and organic phases organize and connect at the microscopic level. According to Bini et al. (2021), by modeling these fibrils in 3D, percolation networks describe the transition from a state where mineral particles are isolated to one where they form a continuous network within the collagen matrix. This approach enables the analysis of how the distribution and concentration of minerals influence the mechanical and functional properties of bone, providing deeper insights into its hierarchical structure and its relationship to strength and flexibility. Moreover, applying percolation theory in this context helps identify critical mineralization thresholds necessary to achieve effective connectivity, which is essential for developing biomimetic materials and strategies for bone regeneration [69]. The basic idea of percolation theories in some models is that cortical bone is composed of an assembly of nearly cylindrical structures known as osteons. These osteons are made up of mineralized lamellae and collagen fibers encircling the Haversian canal. They are primarily oriented along the longitudinal axis of long bones, with their boundaries defined by cement lines and interstitial bone [70]. From this perspective, cortical bone can be conceptualized as a network of basic structural units represented by osteons, where the connections or boundaries are formed by the cement lines and interstitial tissue. Micro-cracks preferentially progress between osteons, which leads to the failure of these connections. This propagation is accompanied by energy release in the form of AE signals. As the applied load on the bone increases, so does the number of fractured connections within the network. A macroscopic fracture occurs when a critical number of connected bonds fail (this is one of the key ideas of percolation theory [67,68]), resulting in the network being completely compromised.

**Figure 4:**
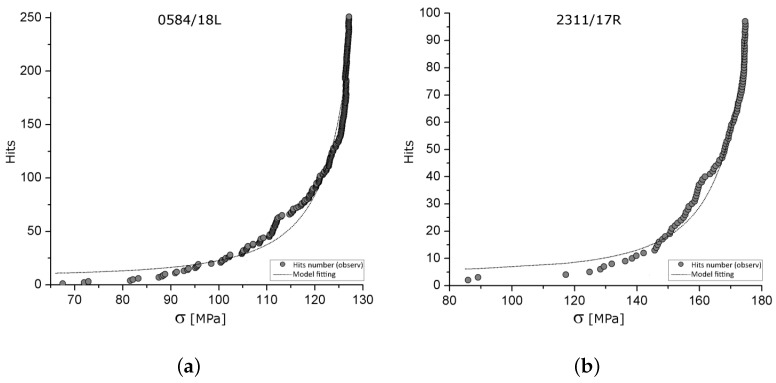
*Hits*-stress plot (dots) of rib bending tests detected by the AE technique and model fitting (line) for two different specimens (**a**,**b**). As in Figure 3, AE signals (hits) are concentrated at high stress levels in a clear vertical asymptote, as predicted by percolation theory.

If we denote $p_{\sigma }$ as the probability of failure of an interosteonic connection (as a function of the principal stress), percolation theory states that there exists a threshold value $p_{c}$, near which the number of broken connections, which would generate AE signals, is given by [70]

(4) $$N_{AE}\propto \frac{1}{|p_{\sigma }-p_{c}{|}^{\gamma }}={\left|\left(1-p_{c}\right)-\exp\left[{\left(\frac{\sigma }{\sigma_{\infty }}\right)}^{\alpha }\ln\left(1-p_{c}\right)\right]\right|}^{-\gamma }$$

where γ is a *universal critical exponent* (independent of the shape of the osteon network), under the assumption that $p_{\sigma }$ follows a probability distribution as predicted by extreme value theory [65]. Parameters $p_{c}$ and α are determined based on percolation theory ($p_{c}\approx 1/2$) and brittle and bone material experiments (α≈4) [70]. Thus, Equation (4) predicts the expected number of AE signals ($N_{AE}$) for each stress level σ, where both quantities can be experimentally measured, allowing for the values of γ and $\sigma_{\infty }$ to be fitted (see Figure 4).

## 5. Conclusions

The implementation of acoustic emission in bone tissue has shown that this technique can be very useful in different fields, such as the detection of the onset of bone damage, the evaluation of healing after treatment, or the study of the presence of bone pathologies. Although several studies have used acoustic emission in bone, most of them have been used to evaluate the level of stress at which failure begins, to discern between the regions of mechanical behavior of the bone, or to evaluate the influence of variables such as load rate, anisotropy, or porosity. However, there is a large gap in the study of the relation between the intrinsic mechanical properties of bone tissue and the characteristics of AE, which is of great relevance in order to understand how bone behaves from the use of this technique. Likewise, there are very few studies that have used acoustic emission to develop predictive models that allow for advancing the macroscopic failure of the bone from EA.

The application of AE in bone research has some limitations. Accurate localization based on the time difference of arrival becomes unreliable in the presence of anisotropies or density variations. Moreover, most AE studies do not utilize intrinsic mechanical measures, such as stress or strain, that are not independent of geometry. Experimentally, securing sensors to specimens is a significant challenge, as proper contact is essential for reliable event detection. Data interpretation is further complicated by the lack of signal classification, which makes it impossible to identify the type of micro-failure associated with a specific AE signal. While such classification methods have been applied to other materials, no studies have yet explored their use in bone.

## Figures and Tables

**Table 2:** Influence of mineralization, anisotropy, load rate, and porosity on AE characteristics.

Property	Trend
Mineralization [7,34] (higher)	- Less AE events - Higher AE energy - Lower stress and strain to failure
Anisotropy [7]	- Transverse → Higher cumulative energy - Transverse → Higher AE energy - Transverse → AE at lower strain
Porosity (pore size) [48] (higher)	- Higher AE activity
Strain rate [11,49] (higher)	- Lower number of AE events - Higher AE amplitude - Damage at lower load

**Table 3:** Models comparison.

Model	Type	Outcomes	Drawbacks
Risk curves [44,45,52]	Probabilistic	Failure probability	- Non precise prediction - Depends on specimens number
Percolation [67,68,69,70]	Semi-probabilistic	Approximateultimate stress	- Reliable near ultimate stress - Unable to provide error margins
Stochastic breaking [62,65]	Stochastic	Probabilitydistribution	- Non deterministic values - Provides information on variation

## Data Availability

Not applicable. This a review article and no new data were used for this investigation.

## Appendix A. Inclusion and Exclusion Criteria

This appendix provides a comprehensive explanation of the phases involved in selecting the works reviewed, detailing how the inclusion and exclusion criteria were progressively refined:

- *Search*: the database Scopus was used to search for all documents including the words “acoustic”, “emission”, and “bone”, obtaining 522 results. From these documents, the database was used to include only those:
  − English language. Chinese (13), German (7), Japanese (6), Russian (2), or from other languages (13) were excluded, reducing the initial collection to 481 documents.
  − Article o review types. Conference papers (76) or conference reviews (12), book chapters (6), and other types of documents (8) were excluded, leaving 379 articles and reviews remaining.
- *Screening*: the 379 considered documents were filtered in two stages; first automatically based on specific frequent words that are not inside the scope of this article (this first stage was manually reviewed), and second manually to exclude other articles not related.
  − First stage: words “ear”, “hearing”, and “hear” were used to filter the data, and those articles inside this filter were manually reviewed (185). The resulting list was limited to 194.
  − Second stage: the abstracts of the remaining 194 documents were read. Those articles falling outside the scope, with topics related to otoacoustics or hearing (7), surrogates or materials other than bone (59), illnesses, pathologies, prostheses, coatings, implants or therapies (28), bone machining (13), use of other techniques as radiation, cavitation, vibroacoustics (18), or not directly related to bone mechanical properties or modeling (5) were excluded. A total of 64 documents were included.
- *Inclusion*: The remaining 64 documents were read. From those, seven articles were not considered, as they were not inside the scope. Finally, 57 articles were included in this review, which dealt with AE and its mechanical performance on bone, or AE modeling in bone.
